# Prevalence and Mortality of Moderate or Severe Mitral Regurgitation Among Patients Undergoing Percutaneous Coronary Intervention With or Without Heart Failure: Results From CIN Study With 28,358 Patients

**DOI:** 10.3389/fcvm.2022.796447

**Published:** 2022-03-03

**Authors:** Haozhang Huang, Jin Liu, Kunming Bao, Xiaoyu Huang, Dehua Huang, Haiyan Wei, Nuerbahaer Remutula, Tilakezi Tuersun, Wenguang Lai, Qiang Li, Bo Wang, Yibo He, Heyin Yang, Shiqun Chen, Jiyan Chen, Kaihong Chen, Ning Tan, Xiaoyan Wang, Liling Chen, Yong Liu

**Affiliations:** ^1^Department of Guangdong Provincial Key Laboratory of Coronary Heart Disease Prevention, Guangdong Cardiovascular Institute, Guangdong Provincial People's Hospital, Guangdong Academy of Medical Sciences, Guangzhou, China; ^2^Department of Cardiology, Longyan First Hospital Affiliated With Fujian Medical University, Longyan, China; ^3^People's Hospital of Yangjiang, Yangjiang, China; ^4^Department of Cardiology, First People's Hospital of Kashgar, Kashgar, China; ^5^Guangdong Provincial People's Hospital, School of Medicine, South China University of Technology, Guangzhou, China; ^6^The First Affiliated Hospital, Sun Yat-sen University, Guangzhou, China; ^7^Department of Nuclear Medicine, The First Affiliated Hospital of Sun Yat-sen University, Guangzhou, China

**Keywords:** moderate or severe mitral regurgitation, percutaneous coronary intervention, heart failure, coronary artery disease, prevalence, mortality

## Abstract

**Aim:**

This study investigated the prevalence and mortality associated with moderate or severe mitral regurgitation (MR) among patients undergoing percutaneous coronary intervention (PCI), with or without heart failure (HF).

**Methods:**

We analyzed patients undergoing PCI without mitral valve surgery from the Cardiorenal ImprovemeNt (CIN) study (ClinicalTrials.gov NCT04407936). Patients without echocardiography to determine MR occurrence or lacking follow-up death data were excluded. Primary endpoints were 1-year and long-term all-cause mortality, with a median follow-up time of 5 years (interquartile range: 3.1–7.6).

**Results:**

Of 28,358 patients undergoing PCI treatment [mean age: 62.7 ± 10.7; women: 6,749 (25.6%)], 3,506 (12.4%) had moderate or severe MR, and there was a higher rate of moderate or severe MR in HF group than non-HF group (28.8 vs. 5.6%, respectively). Regardless of HF conditions, patients with moderate or severe MR were older and had worse cardio-renal function and significantly increased 1-year mortality [adjusted hazard ratio (aHR): 1.82, 95% confidence interval (CI): 1.51–2.2], and long-term mortality [aHR: 1.43, 95% CI: 1.3–1.58]. There was no significant difference between patients with HF and those with non-HF (*P* for interaction > 0.05).

**Conclusion:**

One-eighth of the patients undergoing PCI had moderate or severe MR. Furthermore, one-third and one-seventeenth experienced moderate or severe MR with worse cardiorenal function in the HF and non-HF groups, and increased consistent mortality risk. Further studies should explore the efficacy of mitral interventional procedures for moderate or severe MR after PCI treatment, regardless of HF.

## Introduction

Mitral regurgitation (MR) is a common valvular disease and serves as a worse prognosis predictor, especially in patients with coronary artery disease (CAD) (1–3), and up to 50% of moderate or severe MR pathological remodeling was attributed to chronic CAD (4).

Percutaneous coronary intervention (PCI) has become the most common revascularization strategy for patients with obstructive CAD; it can reduce the area of myocardial ischemia and reflux of MR (5, 6). There were several studies indicating a lower survival rate of moderate or severe MR among patients undergoing PCI, but these were limited to small samples or CAD subtypes (7, 8). Therefore, large-scale cohort studies on prevalence and outcomes of moderate or severe MR among patients with CAD undergoing PCI are still lacking.

With improvement in management, the treatment rate of PCI is increasing in patients with CAD with HF, but the prognosis remains poor because of complicated clinical features (9). Previous studies have demonstrated that moderate or severe MR is common among patients with HF and associated with poor prognosis (10–12), while the role of moderate or severe MR in patients with HF undergoing PCI has been poorly addressed. Furthermore, whether there is higher incidence and mortality of moderate or severe MR among PCI with HF compared to those without has not been previously reported.

To address some of these knowledge gaps, we aimed to systematically explore the prevalence and outcomes of moderate or severe MR compared with normal or mild MR among patients with CAD undergoing PCI. Most importantly, we intended to test the hypothesis that these patients, with or without HF, would have significant differences in mortality risk in moderate or severe MR.

## Methods

### Study Design and Patient Selection

**Figure 1:**
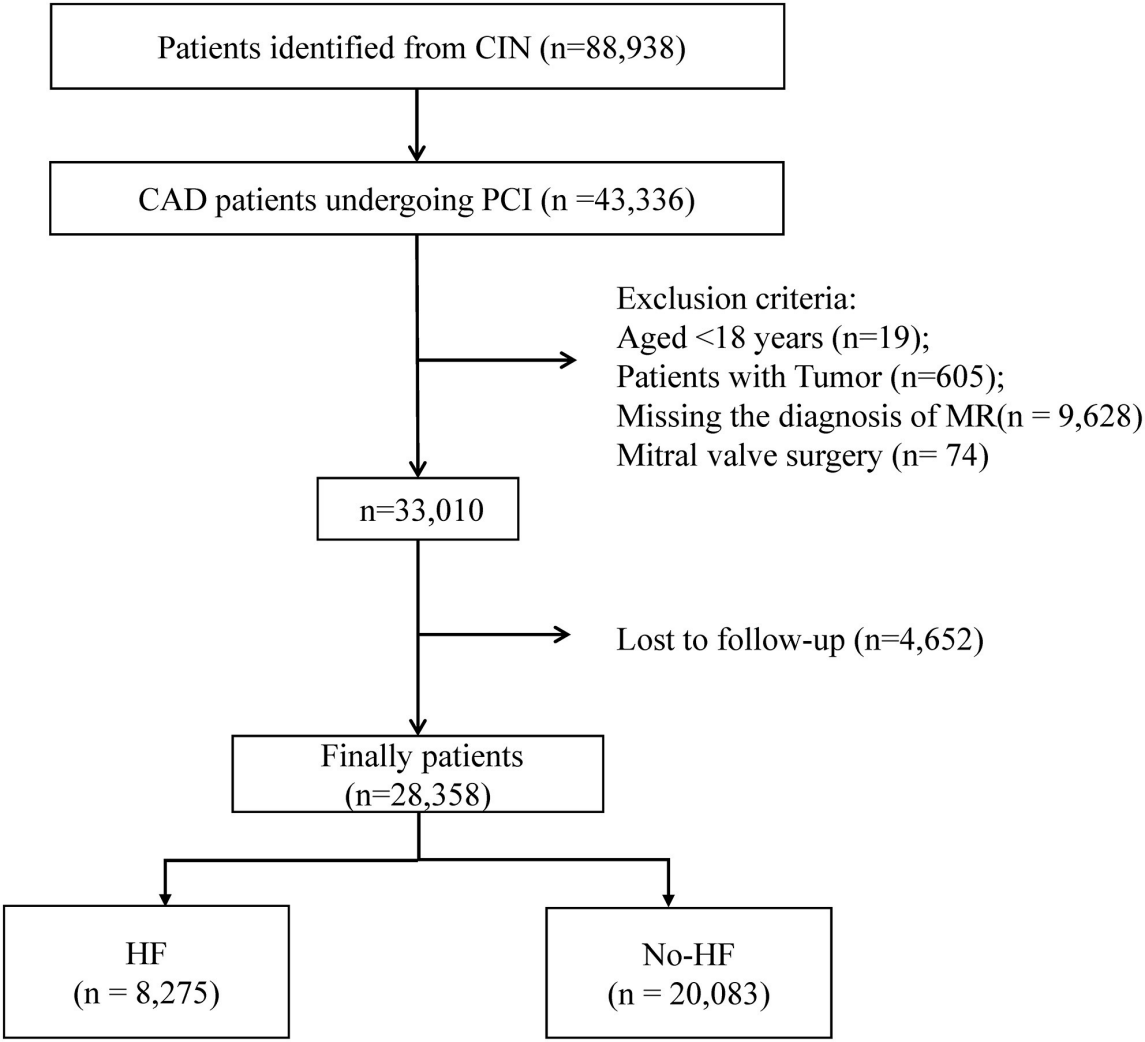
Flow of participants through the trial.

The Cardiorenal ImprovemeNt (CIN) Registry is a retrospective, single-center, observational cohort study that enrolled patients undergoing PCI treatment according to standardized clinical practice guidelines at Guangdong Provincial People's Hospital, China, from January 2007 to December 2018 (ClinicalTrials.gov NCT04407936) (13, 14). Among these patients, 8,275 were complicated by HF. The exclusion criteria were (1) age <18 years (*n* = 12); (2) life expectancy <1 year due to malignancy or other end-stage diseases (*n* = 346); (3) subsequent mitral valve surgery (*n* = 74); and (4) lack of follow-up data (*n* = 3,235). Finally, 28,358 patients undergoing PCI treatment complicated by MR, with or without HF, were included in our study (Figure 1). The study population was divided into four groups according to MR severity and cardiac function as follows: Group 1 experienced HF and was classified as normal or mild MR; Group 2 experienced HF with moderate or severe MR; Group 3 did not experience HF (non-HF) and was classified as normal or mild MR; Group 4 did not experience HF with moderate or severe MR.

### Data Extraction

The presence of MR was determined according to the results of first echocardiography examination, and the severity of MR was derived from the echocardiogram report and classified according to two levels (normal or mild vs. moderate or severe). MR severity was evaluated by visual assessment integrating Doppler data from multiple acoustic windows and incorporating qualitative and semi-quantitative methods. Senior echocardiography physicians were responsible for data quality control and periodic database verification.

### Outcomes and Definitions

The primary endpoints were 1-year and long-term all-cause mortality. The follow-up data were obtained from the Guangdong Provincial Public Security database, which was matched against the electronic Clinical Management System of the Guangdong Provincial People's Hospital records based on the unique ID number for each patient.

Heart failure (HF) status was assessed according to signs, symptoms, and guideline-based laboratory tests (15, 16). Comorbidities included hypertension (HT), diabetes mellitus (DM), acute myocardial infarction (AMI), atrial fibrillation(AF), chronic kidney disease (CKD, defined as eGFR ≤ 60 ml/min/1.73 m^2^), anemia (defined as hematocrit <36% for women and <39% for men) (17), hyperlipidemia (defined according to 2016 ESC guidelines for treating dyslipidemias) (18), and chronic obstructive pulmonary disease.

### Statistical Analysis

Descriptive statistics for baseline variables are presented as the mean [standard deviation (SD)], median [interquartile range (IQR)], or number and percentage as appropriate. Differences in baseline characteristics between two groups were analyzed by Student's *t*-test and Pearson's Chi-squared test. Comparison among multiple groups was assessed by analysis of variance (ANOVA) (for continuous variables) and chi-square test (for categorical variables) as appropriate. Type I errors were minimized using the Bonferroni correction (Bonferroni correction = 0.05/6).

Kaplan–Meier (KM) analyses with stratified log-rank tests were performed to assess survival among the four groups. A Cox proportional hazards model with multivariable analysis was used to compare 1-year and long-term all-cause mortality risk according to the prevalence of moderate or severe MR among the HF and non-HF groups. Variables known to be associated with mortality according to clinical experience were controlled further by multivariable Cox regression using different models. Model 1 was unadjusted; Model 2 was adjusted for age and sex; and Model 3 included age, gender, hypertension, CKD, AMI, stroke, AF, DM, hyperlipidemia, anemia, in-hospital dialysis, angiotensin-converting enzyme inhibitors (ACEI)/angiotensin receptor blockers (ARB), β-blockers, statins, antiplatelet, calcium channel blocker, mineralocorticoid receptor antagonists (MRA), loop diuretics, and oral anticoagulants.

We also performed eight pre-specified subgroup analyses to assess the effects of moderate or severe MR on long-term all-cause mortality among patients undergoing PCI with or without HF, including male vs. female; age > 65 years vs. ≤ 65 years; AMI vs. non-AMI; and left ventricular dysfunction (LVD) vs. non-LVD. All statistical tests were two-sided, and a threshold of *p*-value < 0.05 was set for significance. All the statistical analyses were performed using R v 4.0.3 (R Institute for Statistical Computing, Vienna, Austria).

## Results

### Baseline Characteristics

#### Overall Characteristics of the Whole Population

From January 2007 to December 2018, a total of 28,358 PCI patients were enrolled in the final analysis [mean age: 62.6 ± 10.7 years, 6,749 (23.8%) women], and 53.9% of patients undergoing PCI suffered from MR. The prevalence of moderate or severe MR was 12.4% (*n* = 3,506). A total of 8,275 patients undergoing PCI experienced HF [mean age: 63.04 ± 11.1 years, 2,029 (24.5%) were women].

#### Baseline Characteristics of Patients Undergoing PCI With HF

Overall, among the patients undergoing PCI with HF, 28.8% (*n* = 2,386) were classified as experiencing moderate or severe MR (Table 1). Patients with moderate or severe MR were more likely to be older (*P* < 0.001), and the prevalence of complications increased, such as anemia, CKD, and atrial fibrillation (*P* < 0.001) compared with the normal and mild MR groups. In contrast, the prevalence of hypertension, stroke, COPD, hyperlipidemia, and previous coronary artery bypass graft (CABG) did not differ significantly among the different groups. Higher prevalence of prior PCI was reported among the patients with moderate or severe MR (*P* = 0.08) compared with the normal and mild MR groups. Higher prevalence of DM was also observed among patients undergoing PCI with moderate or severe MR compared with the normal and mild MR groups.

**Table 1:** Baseline characteristics of the patients undergoing percutaneous coronary intervention (PCI) with different levels of mitral regurgitation severity stratified by heart failure (HF).

**Characteristic**	**HF**				**Non-HF**				*P*-value^*^
	**Overall**	**Normal/mild**	**Moderate/severe**	***P*-value**	**Overall**	**Normal/mild**	**Moderate/severe**	***P*-value**	
	**(*n* = 8,275)**	**(*n* = 5,889)**	**(*n* = 2,386)**		**(*n* = 20,083)**	**(*n* = 18,963)**	**(*n* = 1,120)**		
**Demographic characteristics**
Age, years	63.0 (11.1)	62.5 (11.2)	64.4 (10.8)	<0.001	62.4 (10.6)	62.2 (10.5)	65.6 (10.6)	<0.001	<0.001
Age group, *n* (%)			<0.001				<0.001	<0.001
<60	2,939 (35.5)	2,201 (37.4)	738 (30.9)		7,814 (38.9)	7,497 (39.5)	317 (28.3)		
60–75	4,070 (49.2)	2,865 (48.7)	1,205 (50.5)		9,512 (47.4)	8,970 (47.3)	542 (48.4)		
≥75	1,266 (15.3)	823 (14.0)	443 (18.6)		2,757 (13.7)	2,496 (13.2)	261 (23.3)		
Women, *n* (%)	2,029 (24.5)	1,416 (24.0)	613 (25.7)	0.121	4,720 (23.5)	4,423 (23.3)	297 (26.5)	0.016	0.096
**Medical history**					11,339 (56.5)	10,701 (56.4)	638 (57.0)	0.75	>0.99
Anemia, *n* (%)	3,758 (46.4)	2,547 (44.2)	1,211 (51.9)	<0.001	5,556 (28.8)	5,128 (28.1)	428 (40.1)	<0.001	<0.001
HT, *n* (%)									>0.99
AMI, *n* (%)	3,914 (47.3)	2,996 (50.9)	918 (38.5)	<0.001	3,978 (19.8)	3,613 (19.1)	365 (32.6)	<0.001	<0.001
DM, *n* (%)	2,767 (33.4)	1,913 (32.5)	854 (35.8)	0.004	7,252 (28.0)	6,397 (27.5)	855 (32.4)	<0.001	<0.001
CKD, *n* (%)	2,798 (33.8)	1,805 (30.7)	993 (41.6)	<0.001	2,580 (12.8)	2,347 (12.4)	233 (20.8)	<0.001	<0.001
AF, *n* (%)	333 (4.0)	162 (2.8)	171 (7.2)	<0.001	270 (1.3)	202 (1.1)	68 (6.1)	<0.001	<0.001
Stroke, *n* (%)	592 (7.2)	408 (6.9)	184 (7.7)	0.228	1,047 (5.2)	997 (5.3)	50 (4.5)	0.275	<0.001
COPD, *n* (%)	95 (1.1)	69 (1.2)	26 (1.1)	0.839	130 (0.6)	118 (0.6)	12 (1.1)	0.103	<0.001
Hyperlipidemia, *n* (%)	5,726 (71.3)	4,055 (70.8)	1,671 (72.5)	0.136	13,269 (68.1)	12,533 (68.1)	736 (68.1)	0.986	<0.001
**Laboratory tests**
LDLC, mmol/L	2.88 (0.99)	2.88 (0.99)	2.88 (1.00)	0.996	2.85 (0.98)	2.85 (0.98)	2.86 (0.96)	0.956	>0.99
HDLC, mmol/L	0.96 (0.25)	0.96 (0.25)	0.93 (0.25)	<0.001	0.99 (0.25)	0.99 (0.25)	0.98 (0.26)	0.139	<0.001
CMV, ml	167.6 (78.4)	166.0 (76.5)	171.4 (82.8)	0.006	166.0 (75.8)	165.8 (75.5)	169.5 (80.0)	0.12	0.036
ALB, g/L	34.0 (4.6)	34.3 (4.6)	33.4 (4.6)	<0.001	36.9 (3.9)	37.0 (3.8)	34.9 (4.4)	<0.001	<0.001
eGFR, mL/min/1.73 m^2^	67.8 (27.4)	70.0 (27.7)	62.6 (25.9)	<0.001	81.2 (22.7)	81.5 (22.6)	76.4 (24.4)	<0.001	<0.001
**Cardiac indicators**
ProBNP, pg/ml	1,895 [1,047, 3,932]	1,611 [951, 3,155]	2,906 [1,563, 5,821]	<0.001	134 [54, 339]	126 [52, 319]	413 [208, 669]	<0.001	<0.001
LVEDD, mm	52.7 (8.0)	50.9 (7.2)	57.2 (8.2)	<0.001	46.6 (4.9)	46.4 (4.7)	50.7 (5.8)	<0.001	<0.001
LVESD, mm	38.5 (10.1)	36.2 (9.0)	44.2 (10.4)	<0.001	29.50 (5.27)	29.26 (5.07)	34.14 (6.76)	<0.001	<0.001
LA size, mm	37.8 (6.4)	36.7 (6.1)	40.4 (6.4)	<0.001	35.42 (5.60)	35.21 (5.51)	39.10 (5.73)	<0.001	<0.001
LVEF, %	47.7 (13.3)	50.2 (12.7)	41.6 (12.8)	<0.001	62.9 (7.7)	63.2 (7.6)	57.6 (8.8)	<0.001	<0.001
E/A	0.76 (0.33)	0.73 (0.31)	0.84 (0.39)	<0.001	0.71 (0.26)	0.71 (0.26)	0.77 (0.30)	<0.001	<0.001
**Medications**
ACEI/ARB, *n* (%)	4,426 (55.3)	3,173 (55.5)	1,253 (54.7)	0.519	10,925 (54.7)	10,298 (54.6)	627 (56.5)	0.218	>0.99
Beta-blocker, *n* (%)	6,805 (85.0)	4,915 (86.0)	1,890 (82.5)	<0.001	16,953 (84.9)	16,022 (84.9)	931 (83.9)	0.395	<0.001
CCB, *n* (%)	1,255 (15.7)	925 (16.2)	330 (14.4)	0.052	4,210 (21.1)	3,994 (21.2)	216 (19.5)	0.191	<0.001
Statins, *n* (%)	7,743 (96.8)	5,559 (97.3)	2,184 (95.4)	<0.001	19,601 (98.1)	18,525 (98.2)	1,076 (97.0)	0.006	<0.001
Antiplatelet, *n* (%)	7,973 (99.6)	5,692 (99.6)	2,281 (99.6)	0.999	19,920 (99.7)	18,813 (99.7)	1,107 (99.8)	0.814	>0.99
loop diuretic, *n* (%)	2,635 (32.9)	1,455 (25.5)	1,180 (51.5)	<0.001	821 (4.1)	646 (3.4)	175 (15.8)	<0.001	<0.001
MRA, *n* (%)	2,690 (33.6)	1,501 (26.3)	1,189 (51.9)	<0.001	950 (4.8)	758 (4.0)	192 (17.3)	<0.001	<0.001

*ACEI/ARB, angiotensin-converting enzyme inhibitor/angiotensin receptor blocker; ALB, albumin; AMI, acute myocardial infarction; CCB, calcium channel blocker; CKD, chronic kidney disease; COPD, chronic obstructive pulmonary disease; DM, diabetes mellitus; HT, hypertension; eGFR, estimated glomerular filtration rate; LDL-C, low-density lipoprotein cholesterol; LVEDD, left ventricular end-diastolic dimension; LVESD, left ventricular end-systolic diameter; LA size, left atrial size; LVEF, left ventricular ejection fraction; MRA, mineralocorticoid receptor antagonist; PCI, percutaneous coronary intervention*.

* *Bonferroni correction for multiple comparisons*.

The distribution of cardiac indicators among patients undergoing PCI with HF, stratified by MR severity, was also significantly different. With increase in MR severity, left ventricular end-diastolic dimension (LEVDD), left ventricular end-systolic diameter; LA size (LVESD), left atrial size (LA size), E/A, and N-terminal pro-brain natriuretic peptide (NT-proBNP) gradually increased (*P* < 0.001), whereas left ventricular ejection fraction (LVEF) gradually decreased (*P* < 0.001). In addition, among the patients with HF and moderate or severe MR, ACEI/ARB, beta-blockers, and MRA were used in 54.2, 81.7, and 17.3% of cases, respectively. Among these, beta-blockers were most commonly used in the patients with moderate or severe MR compared with the patients classified as with normal or mild MR.

#### Baseline Characteristics of Patients Undergoing PCI Without HF

A total of 20,083 patients undergoing PCI without HF were enrolled in this study, including 5.6% (*n* = 1,120) diagnosed with moderate or severe MR. Patients undergoing PCI with moderate or severe MR were significantly older and had lower LVEF, and higher LVEDD, LVESD, LA size, and E/A than patients undergoing PCI diagnosed as normal or with mild MR The prevalence of complications, such as DM, anemia, CKD, atrial fibrillation, and AMI (*P* < 0.001), increased among patients with moderate or severe MR compared with those who were normal or with mild MR. In addition, patients diagnosed with moderate or severe MR were more likely to use MRA and loop diuretics compared with those who were normal or with mild MR (Table 1).

### Mortality

#### 1-Year Mortality

**Figure 2:**
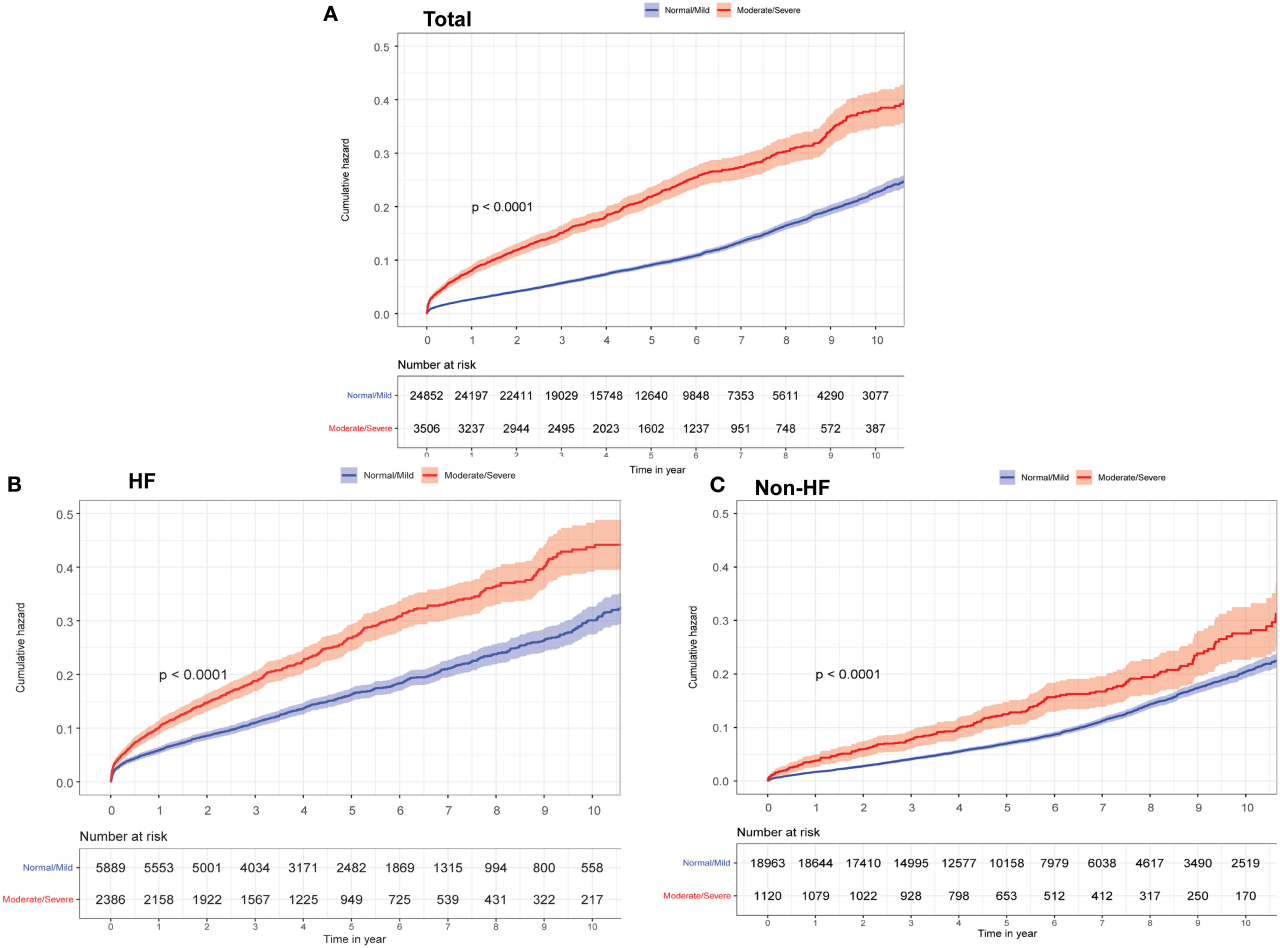
Kaplan-Meier curves for long-term all-cause mortality among different groups according to heart failure (HF) and mitral regurgitation (MR) severity. **(A)** Total. **(B)** HF. **(C)** Non-HF.

During the 1-year follow-up, a total of 360 (1.8%) and 564 (6.8%) patients died from all causes among patients undergoing PCI with and without HF, respectively. As determined by Kaplan–Meier survival curves (Figure 2), moderate or severe MR was associated with increased risk of 1-year mortality among patients who underwent PCI. A greater proportion of patients who underwent PCI with HF had all causes of mortality compared to the other group without HF (9.6 vs. 3.7%).

Among patients undergoing PCI with or without HF, relationships between 1-year all-cause mortality and MR severity were evaluated using Cox proportional hazards models (moderate or severe vs. normal or mild). The results indicated that the patients with moderate or severe MR had higher 1-year all-cause mortality risk (total: adjusted hazard ratio (aHR): 1.82, 95% confidence interval (CI): 1.51–2.2; *P* < 0.001; HF group: aHR: 1.57, 95% CI: 1.26–1.96; *P* < 0.001; non-HF group: aHR: 1.63, 95% CI: 1.11–2.4; *P* = 0.012; *P* for interaction = 0.33; Table 2).

**Table 2:** Cox proportional hazard ratios for 1-year and long-term all-cause mortality in different models.

	Model 1^*^(HR, 95% CI)	***P*-value**	**Model 2^#^ (HR, 95% CI)**	***P*-value**	**Model 3^§^(HR, 95% CI)**	***P*-value**
**1-year all-cause mortality**
Total	2.19 (1.89–2.54)	<0.001	2.02 (1.74–2.34)	<0.001	1.57 (1.34–1.84)	<0.001
Non-HF	1.67 (1.24–2.25)	0.001	1.57 (1.16–2.11)	0.003	1.46 (1.07–1.99)	0.018
HF	1.42 (1.19–1.69)	<0.001	1.34 (1.13–1.60)	0.001	1.26 (1.05–1.52)	0.015
P-interaction	0.36		0.38		0.42	
**Long-term all-cause mortality**
Total	1.80 (1.66–1.96)	<0.001	1.66 (1.53–1.81)	<0.001	1.48 (1.35–1.61)	<0.001
Non-HF	1.38 (1.20–1.60)	<0.001	1.28 (1.10–1.48)	0.001	1.26 (1.08–1.48)	0.003
HF	1.47 (1.32–1.63)	<0.001	1.39 (1.25–1.54)	<0.001	1.31 (1.17–1.46)	<0.001
P-interaction	0.44		0.28		0.48	

* *Unadjusted*.

# *Adjusted for age and gender*.

§ *Adjusted for age, gender, hypertension, CKD, AMI, stroke, AF, DM, hyperlipidemia, anemia, in-hospital dialysis, angiotensin-converting enzyme inhibitors (ACEI)/angiotensin receptor blockers (ARB), β-blockers, statins, antiplatelet, calcium channel blocker, mineralocorticoid receptor antagonists (MRA), loop diuretics, and oral anticoagulants*.

*Central Illustration. Prevalence and prognostic significance of mitral regurgitation in patients undergoing percutaneous coronary intervention with or without heart failure*.

#### Long-Term Mortality

The median follow-up time was 5 years (interquartile range: 3.1–7.6). The long-term prognosis results indicated that moderate or severe MR was positively associated with mortality in patients undergoing PCI with or without HF. Patients with moderate or severe MR were found to experience a nearly 40% increase in mortality risk compared with patients classified as with normal or mild MR (total, aHR: 1.43, 95% CI: 1.3–1.58; *P* < 0.001; HF group, aHR: 1.35, 95% CI: 1.2–1.52; *P* < 0.001; non-HF group, aHR: 1.27, 95% CI: 1.07–1.52; *P* = 0.006; *P* for interaction = 0.81).

### Subgroup Analyses

**Figure 3:**
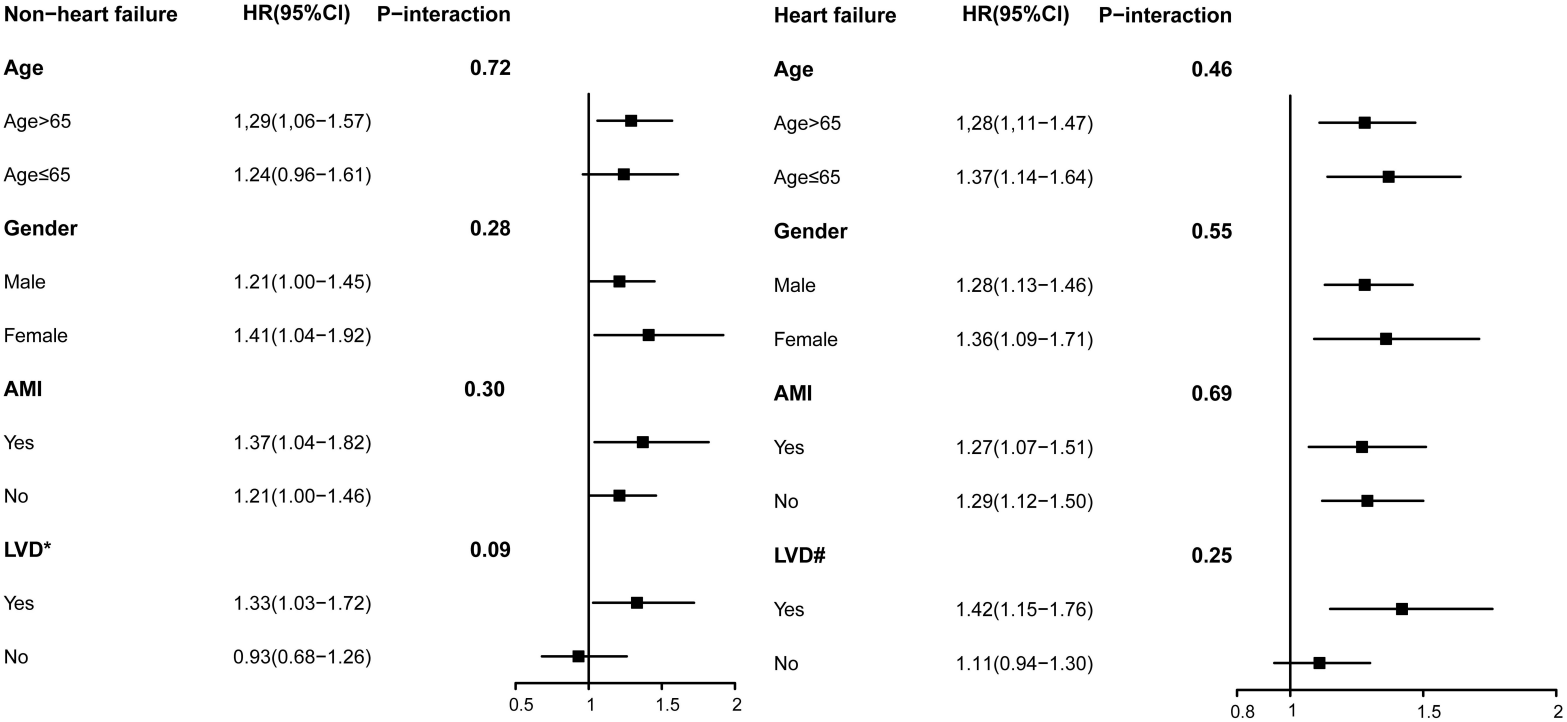
Subgroup analysis of moderate or severe MR among in patients undergoing percutaneous coronary intervention (PCI) without mitral valve surgery. Model 3 for long-term mortality. *LVEF <64(median among non-HF); ^#^LVEF <47(median among HF).

**Figure 4:**
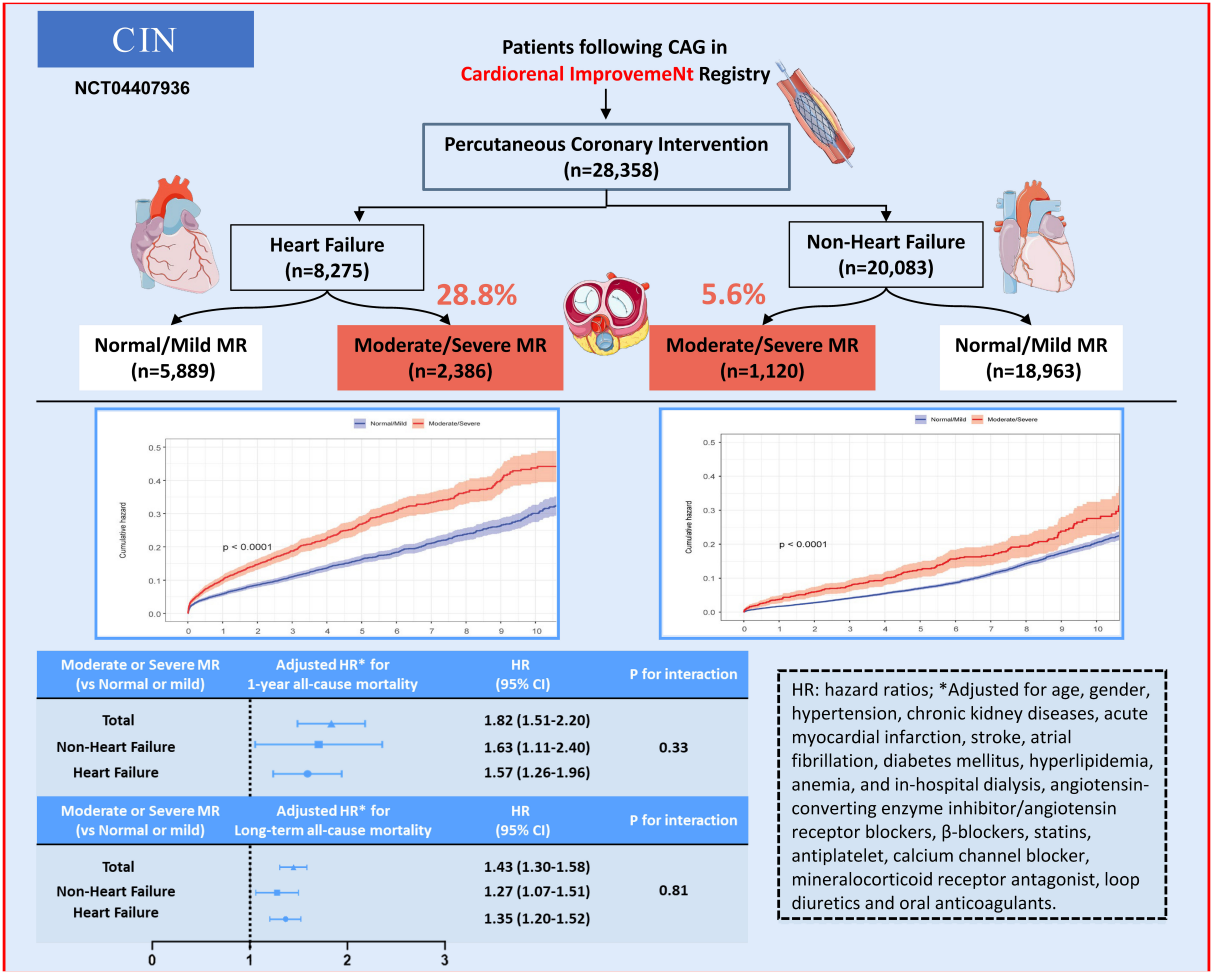
Central illustration.

In the subgroup analyses, Cox regression analysis demonstrated that MR severity was associated with a consistent risk of mortality across dichotomized subgroups, even between the AMI and LVD subgroups (Figure 3). A summary of this study is shown in the central illustration (Figure 4).

## Discussion

To our knowledge, this is the first large cohort study to systematically identify the prevalence and mortality of moderate or severe MR among patients undergoing PCI without mitral valve surgery. Our data showed that moderate or severe MR was common among patients undergoing PCI (1/17 and 1/3 of patients without and with HF, respectively). Additional risks of 1-year and long-term mortality of ~80 and 40%, respectively, were attributable to moderate or severe MR among patients undergoing PCI with or without HF.

Coronary artery disease (CAD) remains a major clinical and public health challenge, with a huge economic burden worldwide (2, 19), and PCI has become the main strategy for the treatment of obstructive CAD (20, 21). Pastorius et al. indicated that patients with MR undergoing PCI have significantly decreased survival rates, while this study was limited by a small sample, with only 711 patients (7). Uddin et al. indicated that higher grades of MR in 4,005 patients with STEMI undergoing primary PCI are associated with worse short- and long-term outcomes, but they only analyzed patients with STEMI (8). Currently, large-scale cohort studies on prevalence and outcomes of moderate or severe MR among patients with CAD undergoing PCI without mitral valve surgery are still lacking. Several previous cohort studies have also reported an association between MR and mortality among patients with HF (10, 22, 23).

An article published by the Journal of the American College of Cardiology (JACC) demonstrated that moderate or severe MR was not independently associated with 1-year mortality among patients with acute decompensated HF (ADHF) who had LVEF ≥ 50% (12). Another study demonstrated that MR has a negative effect on prognosis only in patients with severely reduced LVEF (11). The relationship between MR and PCI with or without HF has not been fully clarified. Therefore, we hypothesized that MR negatively impacts prognosis only in patients with HF but not in those without HF.

However, in our study, contrary to our hypothesis, moderate or severe MR was an independent risk factor in patients with and without HF. Several possible mechanisms may underlie the relationship between MR and PCI with or with HF. MR in patients with HF and left ventricular dilation occurs because of distortion of the valve apparatus, including apical and posterior displacement of the papillary muscles and annular dilation, which may lead to incomplete closure of the mitral leaflets (22, 24). In addition, the destructive influence of CAD on left ventricle function is well-known, and patients with CAD may experience improved left ventricular function after PCI treatment, which might explain why moderate or severe MR increased the mortality risk of patients undergoing PCI, regardless of HF prevalence. Moderate or severe MR may also increase the risk of poor prognosis independent of HF and other important survival predictors because of increased LV filling pressure, activation of neurohumoral systems, and cellular modifications (25). The relevant mechanisms require further study.

The 2021 European Society of Cardiology (ESC) guidelines for the management of secondary MR in patients with HF recommended that patients undergoing PCI with HF complicated by moderate or severe MR should be considered for further treatment to improve the current poor prognosis (12). However, current CAD management guidelines do not provide convincing evidence in patients undergoing PCI with or without HF (26–30).

In conclusion, we report that moderate or severe MR may increase the risk of poor prognosis, independent of HF occurrence and other important predictors of survival. This finding supports clinicians in the utilization of more aggressive treatments for moderate or severe MR among patients who undergo PCI, even in the absence of HF. These findings also provide an avenue for further improvements in existing guidelines. However, we were unable to establish a causal relationship in the present analysis because of the observational nature of this study. Further studies remain necessary to confirm our findings and to better understand the mechanisms that underlie the association between moderate or severe MR and mortality among patients undergoing PCI with or without HF.

Our investigation is not without limitations. First, the data were extracted from a single-center retrospective study, which hampered our ability to control confounders in the analyses; however, sizeable quantities of the data extracted from medical records allowed us to control some confounders. Second, all of the patients included in the study were from Guangdong Provincial People's Hospital, which represents the largest cardiovascular medical center in South China, and more than half of the subjects were referred from non-teaching and community hospitals in both urban and rural areas. Third, we used echocardiography data from 1-year follow-up without regular monitoring of dynamic changes in MR, which may be important. However, our admission ultrasound was performed by professional cardiac ultrasound experts with a small measurement bias. Fourth, information on cause-specific death was not available in this study, and examining correlations between MR and cause-specific death was difficult. Finally, although we excluded baseline surgical or percutaneous approach of MR, we could not analyze the influence of MR evolution and subsequent surgical or percutaneous approach because of the absence of follow-up data. The above variables are very meaningful for the analysis and interpretation of the results, and we will further collect and analyze the above variables in future studies.

Our cohort suggested that moderate or severe MR was a common event among patients undergoing PCI without mitral valve surgery, with one-third of all the patients experiencing HF and 1-17th of all the patients without HF experiencing moderate or severe MR. Patients with moderate or severe MR were more likely to be older and had worse cardio-renal function; and moderate or severe MR was associated with an over 40% increase in long-term mortality among patients undergoing PCI, regardless of HF occurrence. Our findings supported the idea of conducting further studies to test interventional procedures for moderate or severe MR during PCI, regardless of HF.

## Data Availability Statement

The original contributions presented in the study are included in the article/supplementary materials, further inquiries can be directed to the corresponding authors.

## Ethics Statement

The studies involving human participants were reviewed and approved by Guangdong Provincial People's Hospital Ethics Committee. The Ethics Committee waived the requirement of written informed consent for participation.

## Author Contributions

YL, LC, NT, KC, JC, and SC: designed the study. SC, HY, YH, and BW: collected and reviewed the clinical and laboratory data. WL, TT, QL, and NR: analyzed the data. SC, HH, HW, and DH: performed the statistical analysis. HH, KB, JL, and XH: drafted or revised the manuscript. YL, LC, NT, KC, and JC: reviewed, interpreted, and checked the clinical data. All authors contributed to the article and approved the submitted version.

## Funding

This study was supported by grants from the Natural Science Foundation of Fujian Provincial Science and Technology Department (2018J01405 and 2019J01617). The study was not funded by any industry sponsors.

## Conflict of Interest

The authors declare that the research was conducted in the absence of any commercial or financial relationships that could be construed as a potential conflict of interest.

## Publisher's Note

All claims expressed in this article are solely those of the authors and do not necessarily represent those of their affiliated organizations, or those of the publisher, the editors and the reviewers. Any product that may be evaluated in this article, or claim that may be made by its manufacturer, is not guaranteed or endorsed by the publisher.
